# Shift of the reaction equilibrium at high pressure in the continuous synthesis of neuraminic acid

**DOI:** 10.3762/bjoc.18.59

**Published:** 2022-05-20

**Authors:** Jannis A Reich, Miriam Aßmann, Kristin Hölting, Paul Bubenheim, Jürgen Kuballa, Andreas Liese

**Affiliations:** 1 Institute of Technical Biocatalysis, Hamburg University of Technology, Denickestr. 15, 21073 Hamburg, Germany; 2 GALAB Laboratories GmbH, Am Schleusengraben 7, 21029 Hamburg, Germany

**Keywords:** aldolase, continuous fixed-bed reactor, enzyme, epimerase, GlcNAc, high pressure, immobilization, ManNAc, Neu5Ac, pyruvate

## Abstract

The importance of a compound that helps fight against influenza is, in times of a pandemic, self-evident. In order to produce these compounds in vast quantities, many researchers consider continuous flow reactors in chemical industry as next stepping stone for large scale production. For these reasons, the synthesis of *N*-acetylneuraminic acid (Neu5Ac) in a continuous fixed-bed reactor by an immobilized epimerase and aldolase was investigated in detail. The immobilized enzymes showed high stability, with half-life times > 173 days under storage conditions (6 °C in buffer) and reusability over 50 recycling steps, and were characterized regarding the reaction kinetics (initial rate) and scalability (different lab scales) in a batch reactor. The reaction kinetics were studied in a continuous flow reactor. A high-pressure circular reactor (up to 130 MPa) was applied for the investigation of changes in the position of the reaction equilibrium. By this, equilibrium conversion, selectivity, and yield were increased from 57.9% to 63.9%, 81.9% to 84.7%, and 47.5% to 54.1%, respectively. This indicates a reduction in molar volume from *N*-acetyl-ᴅ-glucosamine (GlcNAc) and pyruvate (Pyr) to Neu5Ac. In particular, the circular reactor showed great potential to study reactions at high pressure while allowing for easy sampling. Additionally, an increase in affinity of pyruvate towards both tested enzymes was observed when high pressure was applied, as evidenced by a decrease of *K*_I_ for the epimerase and *K*_M_ for the aldolase from 108 to 42 mM and 91 to 37 mM, respectively.

## Introduction

In times of a pandemic, the importance of substances to enhance the human immune system is self-explanatory. Among them are sialic acids, which are produced and investigated for this reason, as they are found in cell membranes and play an important role in cell adhesion and signaling [1]. They are also studied, for instance, in Covid-19 research [2]. It has been pointed out that derivatives from Neu5Ac can inhibit viral enzymes [3]. Neu5Ac and its production have been described over the past three decades [4], however, no major breakthrough in its synthesis has been achieved so far [5]. Additionally, some reports underline the importance of sialic acids (rather than Neu5Ac in particular), of which Neu5Ac is the most prominent form [6].

**Figure 1:**
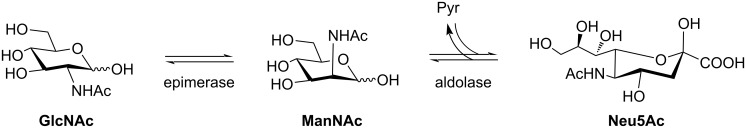
Reaction sequence starting from GlcNAc with ManNAc as an intermediate. Pyr is added in the second step, forming Neu5Ac. The reaction steps are catalyzed by an epimerase (EC 5.1.3.8) and an aldolase (EC 4.1.3.3), respectively.

In 2016, Neu5Ac was approved as a food additive in the United States of America and in the European Union and the Republic of China in 2017 [7]. Due to its importance, Neu5Ac production by enzymatic [5,8] or via whole cell production is still under investigation [9–11]. For this study, the enzymatic synthesis was chosen for its simple reaction sequence (Figure 1) and high selectivity.

Different research groups already described the reaction kinetics of the epimerase and aldolase at ambient pressure [5,8]. In this study, the rate expressions from Groher et al. are used [8]. So far, different approaches have been attempted to increase the overall position of an equilibrium by using additional enzymes [12], different temperatures or high concentrations of the substrates [13].

In accordance with the principle of Le Chatelier, pressure can also be used to influence the position of an equilibrium given that the molar volume changes during the reaction [14]. High-pressure processing is gaining increasing attraction for the enhancement of enzymes [15]. It has been shown that pressure can influence enzymatic reactions, either in kinetics [16–18], in enantiomeric excess [19], in stability [20], or in the position of the equilibrium [17,21]. State of the art for high-pressure research is the use of pressurized batch reactors [22–23].

Since continuous production and suitable reactors are receiving more attention [24], and some believe that this will be the next stepping stone for industry [25–26], the aim of this research was to first establish a continuous reactor at high pressure, and then to investigate the influence of pressure on the reaction sequence to produce Neu5Ac. While high-pressure processes that operate semi-continuously already exist in the food industry, continuous reactors containing a high-performance liquid chromatography (HPLC) pump and a fixed-bed reactor at high pressure are still a relatively new concept. The use of a back pressure regulator up to 10 MPa (100 bar) was already demonstrated by Ötvös et al. [27] or reviewed by Plutschack et al. [28]. In the current work pressures up to 130 MPa were achieved by using an ultrahigh-performance liquid chromatography (UHPLC) pump.

## Results and Discussion

### Immobilization

For the biosynthesis of *N*-acetylneuraminic acid, two enzymes, the epimerase from *Pedobacter heparinus* and the aldolase from *Escherichia coli* K12 were produced in *E. coli* BL21(DE3). Both enzymes were purified and immobilized on different carriers to find for each enzyme the best choice for a stable and active enzyme preparation when applied under high pressure in continuous operation.

For screening purposes, six different carriers were used to immobilize the epimerase and aldolase (Table 1). The carriers differ in their properties (size, hydrophobicity, binding type, and porosity). The quality of immobilization was evaluated in terms of enzyme loading, activity, and reusability in repetitive batch experiments. Furthermore, the most suited carrier with immobilized enzyme was analyzed in long-term studies with respect to the stability of the enzyme preparation.

**Table 1:** List of carriers used for the screening in this work (Lifetech Purolite).

Carrier	Functional group	Binding type	Hydrophobicity	Size [µm]	Pores [Å]

Lifetech ECR8309F amino methacrylate	amino	covalent	hydrophilic	150–300	600–1200
Lifetech ECR8204F epoxy methacrylate	epoxy	covalent	hydrophilic	150–300	300–600
Lifetech ECR8285 epoxy butyl methacrylate	epoxy	covalent	hydrophobic	250–1000	450–650
Lifetech ECR1030M polymethacrylic DVB	none	adsorption	middle	300–710	220–340
Lifetech ECR8806F octadecyl methacrylate	octadecyl	adsorption	hydrophobic	150–300	400–650
Lifetech ECR1090M macroporous styrene	none	adsorption	hydrophobic	300–710	200–300

**Figure 2:**
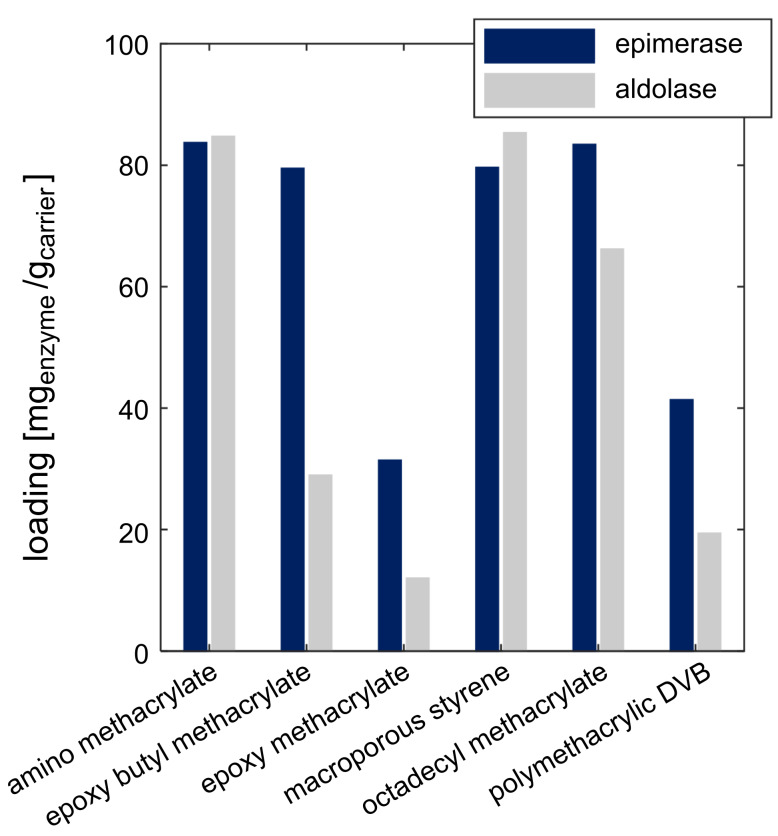
Enzyme loading after immobilization of the epimerase and aldolase on different carriers.

The enzymes were immobilized on six different carriers according to the instructions of the supplier (Lifetech Purolite, Ratingen, Germany). The loading was quantified by analyzing the protein concentration before and after immobilization using the Bradford assay for protein quantification [29]. Screening experiments showed that both enzymes were successfully immobilized on the carriers (Figure 2). For the immobilized epimerase, a maximal loading of 80 mg_enzyme_/g_carrier_ was achieved. Two carriers revealed lower yields with enzyme loadings of 30 and 40 mg_enzyme_/g_carrier_ (epoxy methacrylate, polymethacrylic DVB). The aldolase revealed the highest loadings with 80 mg_enzyme_/g_carrier_ for the amino methacrylate und macroporous styrene carrier. The other analyzed loadings of the aldolase showed a lower yield of 70 mg_enzyme_/g_carrier_ (octadecyl methacrylate) and between 10–30 mg_enzyme_/g_carrier_ for the other evaluated carriers.

**Figure 3:**
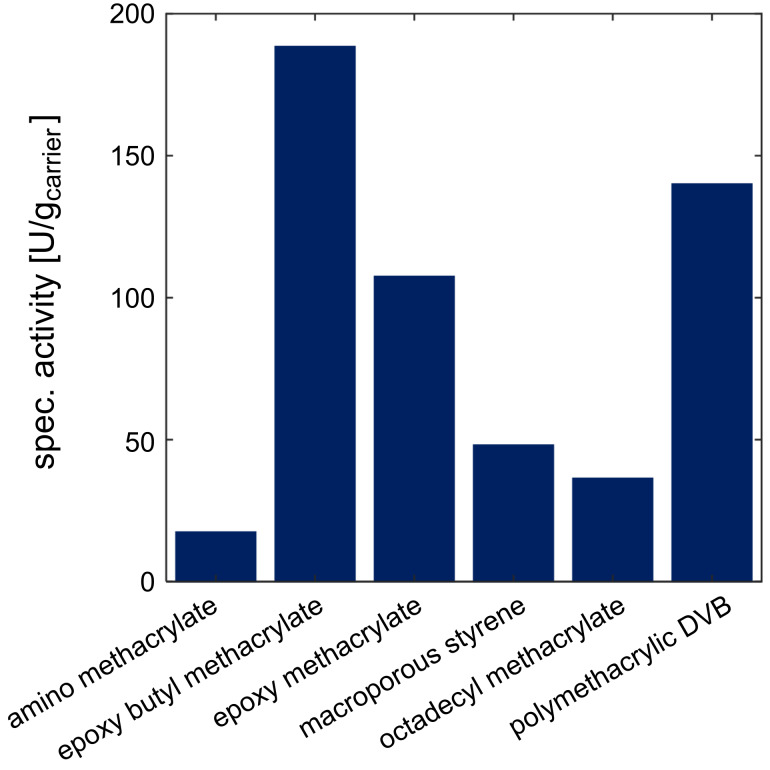
Evaluation of immobilized epimerase on different carriers with respect to specific activity. Reaction conditions: 40 °C, pH 8, 100 mM Tris, U_shaking_ = 1400 rpm, *V* = 1 mL, 100 mM GlcNAc, 1 mM ATP, 1 mM MgCl_2_, immo epimerase 1% (w/v).

**Figure 4:**
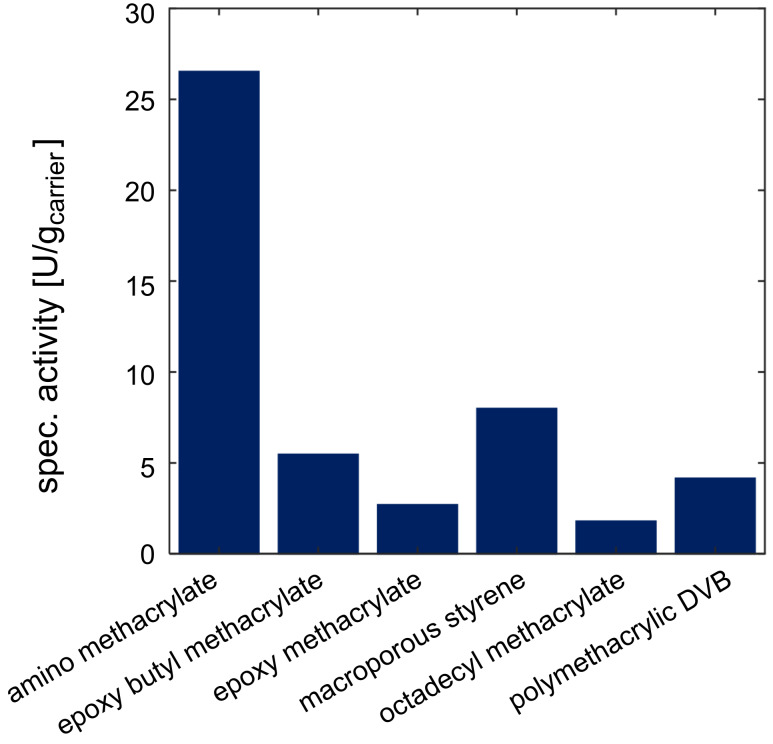
Evaluation of immobilized aldolase on different carriers with respect to specific activity. Reaction conditions: 40 °C, pH 8, 100 mM Tris, U_shaking_ = 1400 rpm, *V* = 1 mL, 100 mM *N*-acetyl-ᴅ-mannosamine, 250 mM pyruvate, immo aldolase 5% (w/v).

The activity of the immobilized enzymes was analyzed in small scale batch experiments with a reaction volume of 1 mL. The carrier was filtered and a defined amount of each carrier was weighed out for the reaction. After the addition of the substrate, samples were taken over the course of time and the specific activity was calculated (Figure 3 and Figure 4).

The product formation of the immobilized epimerase on amino methacrylate reveals the lowest calculated specific activity compared to other utilized carriers with less than 20 U/g_carrier_. The highest specific activity was achieved with two epoxy-functionalized carriers (epoxy butyl methacrylate and polymethacrylic DVB) with over 100 U/g_carrier_. The aldolase reveals the highest activities immobilized on amino methacrylate with about 25 U/g_carrier_. Compared to this, the results of all other specific activities were lower with less than 10 U/g_carrier_. Here the epoxy butyl methacrylate and the macroporous styrene carrier reveal slightly more activity with more than 5 U/g_carrier_ compared to the others with less than 5 U/g_carrier_.

The selection of the most suitable carrier for the immobilization is important for the loading yield of the enzyme on the carrier and the yield of the activity. Both enzymes show the best performance on different materials (epimerase: epoxy butyl methacrylate and aldolase: amino methacrylate). The microenvironment and material surrounding the enzyme have a significant influence on the enzyme activity [30].

**Figure 5:**
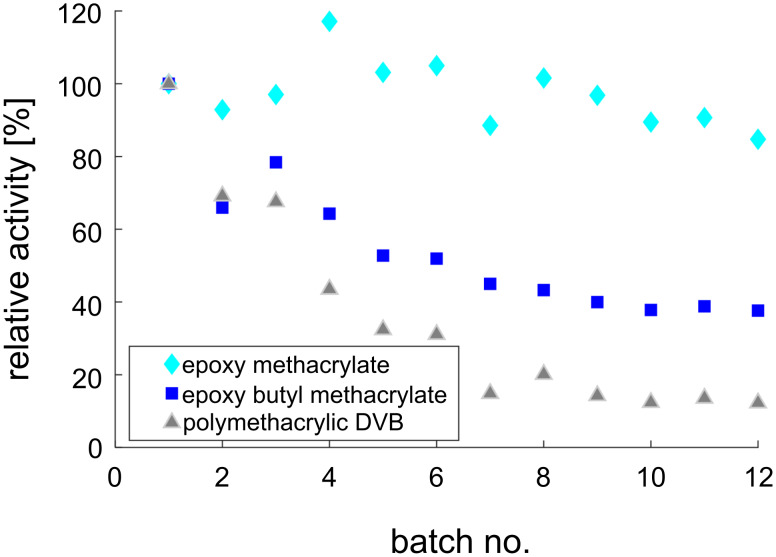
Relative activities in repetitive batch experiments of the immobilized epimerase on polymethacrylate DVB, epoxy methacrylate and epoxy butyl methacrylate. Reaction conditions: 40 °C, pH 8, 100 mM Tris, U_shaker_ = 1400 rpm, *V* = 1 mL, immo epimerase 1% (w/v), 100 mM *N*-acetyl-ᴅ-glucosamine, 1 mM ATP, 1 mM MgCl_2_.

For reusability studies, the three most appropriate carriers were selected and analyzed with respect to the activity of the immobilized enzymes. Reusability was investigated by repetitive batch experiments with up to 5% (w/v) carrier, which is within a range of industrial application of immobilized enzymes in a batch mode [31]. The immobilized epimerase showed in the application in repetitive batches the slightest activity loss using the epoxy methacrylate carrier (Figure 5). For the other analyzed activities on the different carriers, the activity loss was much higher, 60% when using epoxy butyl methacrylate and 90% when polymethacrylate DVB was used.

**Figure 6:**
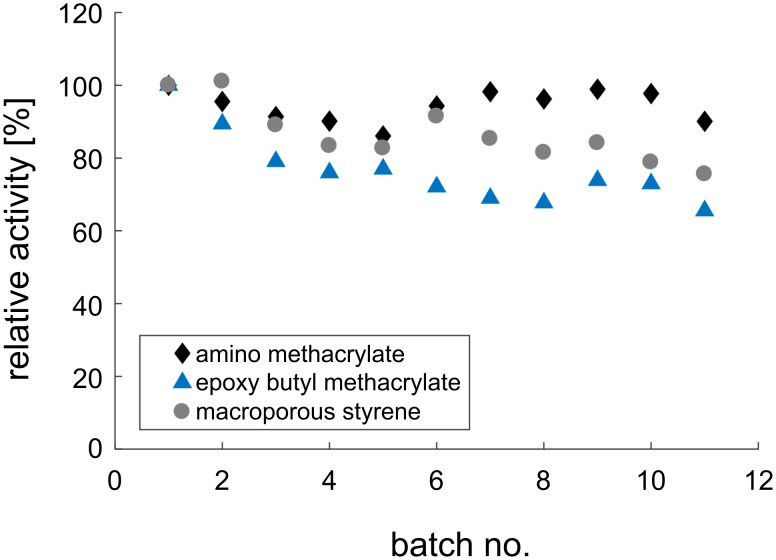
Relative activities in repetitive batch experiments of the immobilized aldolase on amino methacrylate, epoxy butyl methacrylate, and macroporous styrene. Reaction conditions: 40 °C, pH 8, 100 mM Tris, U_shaker_ = 1400 rpm, *V* = 1 mL, immo aldolase 5% (w/v), 100 mM *N*-acetyl-ᴅ-mannosamine, 250 mM pyruvate.

The reusability of the immobilized aldolase was analyzed with amino methacrylate, epoxy butyl methacrylate, and macroporous styrene (Figure 6). All carriers show high suitability for repeated application with the highest loss of activity (35%) for the epoxy butyl methacrylate carrier and macroporous styrene carrier (25%).

**Figure 7:**
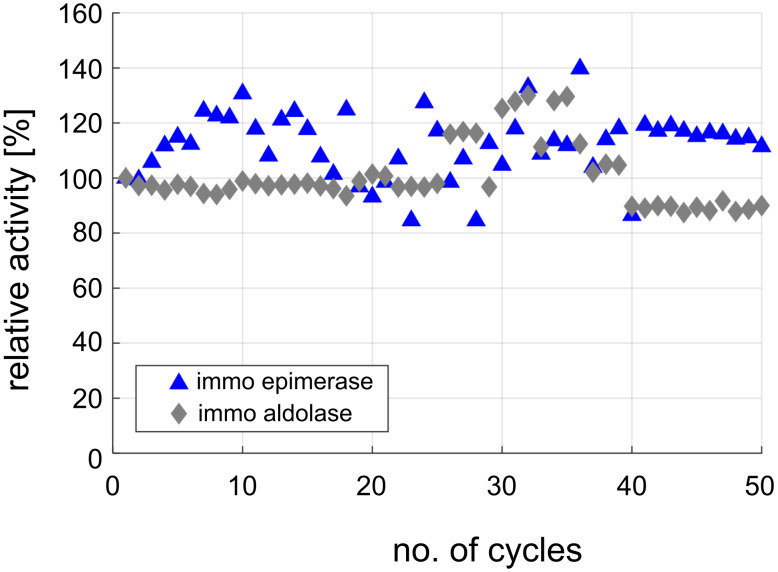
Recycling study of immobilized epimerase and aldolase. Assay conditions: 100 mM Tris, pH 8, 40 °C, shaking with 1000 rpm, *V* = 1 mL. Between the batches the carrier was washed with 100 mM Tris pH 8 and stored until the next application at 4 °C. Reaction conditions: (i) immo epimerase: 100 mM *N*-acetyl*-*ᴅ*-*glucosamine, 1 mM ATP, 1 mM MgCl_2_, 10 g_carrier_/L, loading: 37.6 mg_enzyme_/g_carrier_; (ii) immo aldolase: 100 mM *N*-acetyl*-*ᴅ-mannosamine, 250 mM pyruvate, 70 g_carrier_/L, loading: 95.0 mg_enzyme_/g_carrier_.

Both enzymes showed a suitable reusability in the recycling study. Due to the measured activity, epoxy methacrylate was chosen as carrier for the epimerase and amino methacrylate for the aldolase. The immobilized enzymes were analyzed regarding their reusability over a large number of 50 repetitive batches (Figure 7) and for their stability under storage, and reaction conditions (Table 2).

**Table 2:** Storage, temperature, and mechanic stability of immobilized epimerase and aldolase.^a^

Conditions of stability experiment	immo epimerase τ_1/2_ [d]	immo aldolase τ_1/2_ [d]

6 °C, w/o buffer	87	>179^a^
6 °C, with buffer	>173^a^	>179^a^
40 °C, with buffer	39	58
40 °C, with buffer and shaking	32	46

^a^No significant loss of activity in the analyzed time of the long-term study.

After 50 repetitive batches, both enzymes show almost no loss of activity in the recycling study. The residual activity remains in a range around the initial activity, indicating a high robustness of the selected preparations (Figure 7). The high fluctuation of the relative activity values can be explained by the addition of several measurement errors. Besides the normal standard deviation, a number of other errors affect the results, such as the storage of the immobilized enzymes, the irregularity of carrier washing after the application, the removal of the buffer before application, as well as the sample collection, and sample preparation for the analytics. For further analysis, long-term studies were carried out to analyze the stability in relation to different storage conditions such as the influence of moisture as well as temperature and mechanical stress. The stability of the immobilized enzymes during storage and application is an important criterion for the economic use of the enzyme. Therefore, immobilized enzyme aliquots were stored under four different conditions: filtered and cooled at 6 °C, with buffer at 6 °C, with buffer at 40 °C, and with buffer at 40 °C and shaking at 1000 rpm. During the storage period the activity was measured under standard activity conditions. By calculating of the residual activity, the stability was calculated by exponential fitting [32]. Due to inadequate correlation of the exponential fit to determine the deactivation, a half-life time could not be calculated for all stability tests. In these cases, the loss of activity was minor and a fit by deactivation was not possible. During the experimental period the activity decreased by less than 50%. For the immobilized enzymes stored in buffer with cooling no significant loss of activity was observed during the investigated period of 179 days (immo aldolase) or 173 days (immo epimerase) (Table 2). For the immobilized aldolase, no difference in stability was detected during storage of the filtered carrier with residual moisture or the wet-stored carrier in buffer (>179 days). For the immobilized epimerase, a high loss of stability was observed up to a half-life of about 87 days without buffer, whereas the wet-stored carrier had a half-life of >173 days. The residual moisture of the carrier after filtration is also dependent on the pore sizes. The smaller pore sizes of the 300–600 nm carrier (ECR8204F), used for epimerase immobilization, may not ensure that the enzyme is surrounded by sufficient liquid for an extended storage period. As a result, the activity of the epimerase gets lost. In contrast, almost no loss of activity was observed for the carrier ECR8309F with larger pore sizes (600–1200 nm) that was used for the aldolase immobilization, suggesting that the residual content of moisture significantly influences the stability of the immobilized enzyme. The strongest influence on the stability is evoked by the heating of the immobilized enzymes. A reduction of the half-life time to 39 days or 58 days for the immobilized epimerase and aldolase, respectively, is observed when a continuous temperature exposure is applied. The mechanical stress of shaking the immobilized enzyme decreases the stability by about 20% compared to the reference study without shaking. From the stability investigations, it can be concluded that both selected enzyme preparations have adequate stability for the continuous application under high pressure.

### Kinetics

**Figure 8:**
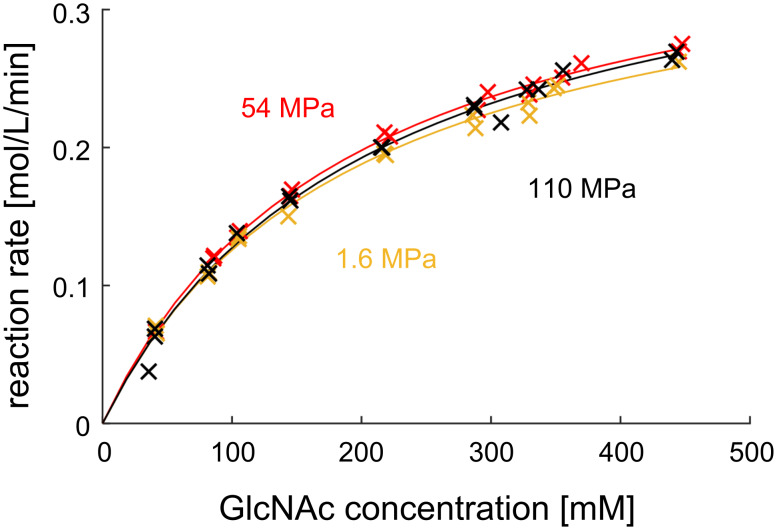
Measured reaction rates of the immobilized epimerase. The dashed line is the fit according to the Michaelis–Menten equation. Conditions: 40 °C, flow rates: 1.6 MPa at 2 mL/min, 54 MPa and 110 MPa at 1.8 mL/min, reactor volume: 0.21 mL, 10 mM potassium phosphate buffer 7.50, 1 mM ATP, 1 mM MgCl_2_, 55 mg particles loaded with epimerase.

Reaction kinetics were first measured for the immobilized epimerase. In the absence of inhibitors or backward reactions, the reaction rate can be modelled as a Michaelis–Menten rate expression. The Michaelis–Menten equation was used to fit the reaction rates at different substrate concentrations for different pressures in Figure 8. The resulting kinetic parameters are listed with 95% confidence intervals in Table 3.

**Table 3:** Kinetic parameters from experiments with immobilized epimerase.

Pressure [MPa]^a^	*K*_M_ [mM]^b^	*a*_sp_ [µmol/g_carrier_/min]^b^	*v*_max_ [mol/L/min]^b^

1.6 ± 0.1	195 ± 26	1324 ± 77	0.37 ± 0.02
54 ± 3.8	208 ± 34	1400 ± 104	0.41 ± 0.03
110 ± 9.3	193 ± 93	1388 ± 47	0.39 ± 0.01

^a^The error given for pressure is the median average difference. ^b^The error given for the kinetic parameters indicates the confidence interval (95%) in the regression.

**Figure 9:**
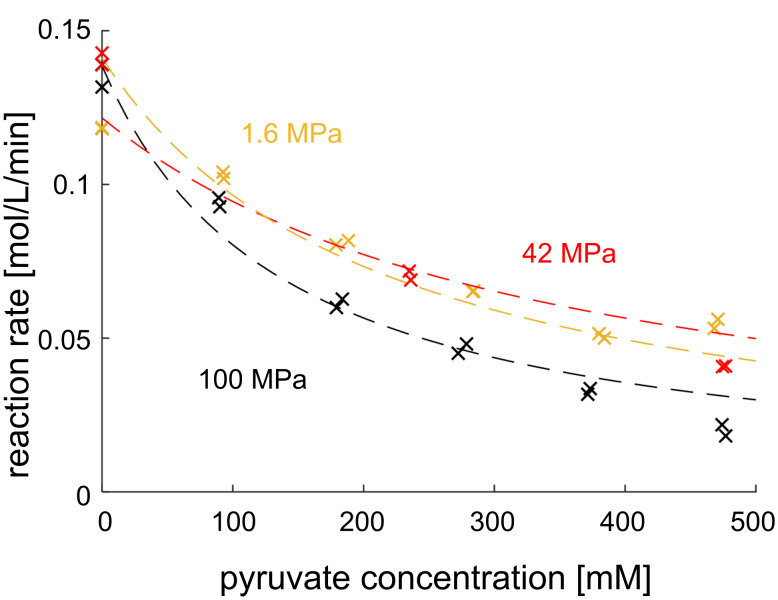
Measured reaction rate of the immobilized epimerase as a function of pyruvate and pressure. Dashed lines are fitted to a competitive inhibition model, 40 °C, volume flow: 2 mL/min, 440 mM GlcNAc, 100 mM buffer, 0.21 mL reactor volume, 2.25 min waited for steady state, 57 mg particles loaded with epimerase.

The influence of pressure on the inhibition by pyruvate was measured and is shown in Figure 9.

**Figure 10:**
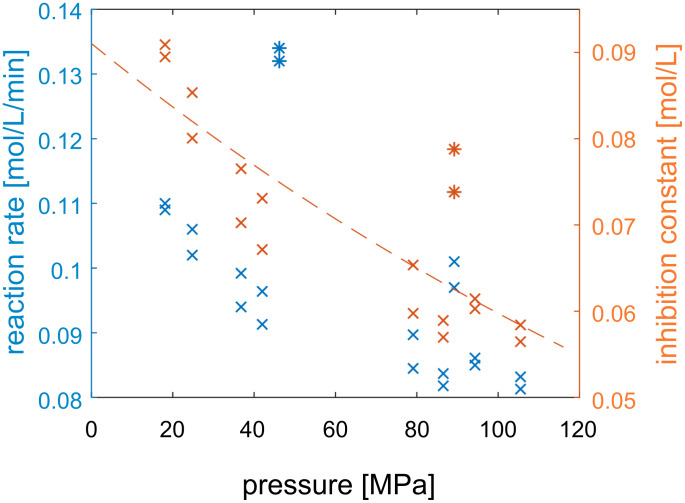
Measured reaction rate (left) and the determined inhibition constant by pyruvate (right) at different pressures. Conditions: 450 mM GlcNAc, 400 mM pyruvate, 2 mL/min, 40 °C, 0.21 mL reactor volume, 57 mg particles loaded with epimerase. Points determined as outliers are marked using asterisks.

The value for the inhibition constant is in the same order of magnitude as results of other groups, measured at ambient pressure (0.146 ± 0.019 mol/L [5]). Since the *K*_I_ value changes with pressure (Table 4), the concentrations were kept constant and only the pressure was varied, resulting in the reactions rates shown in Figure 10 (left). By rearranging the rate expression and inserting the previously calculated kinetic parameters, the inhibition constant was calculated (Figure 10 (right)).

**Table 4:** Determined inhibition constant for pyruvate for the immobilized epimerase.

Pressure [MPa]	*K*_I_ [mM]^a^

1.6	108 ± 21
42	67 ± 11
100	43 ± 10

^a^The error given for the kinetic parameter indicates the confidence interval (95%) in the regression.

The change in molar volume introduced by the coupling of pyruvate to the enzyme was calculated by using the exponential fit. The calculated value is −12.9 ± 5.5 mL/mol (the values at 50 MPa and 89 MPa were considered as outliers and not included in the calculation).

**Figure 11:**
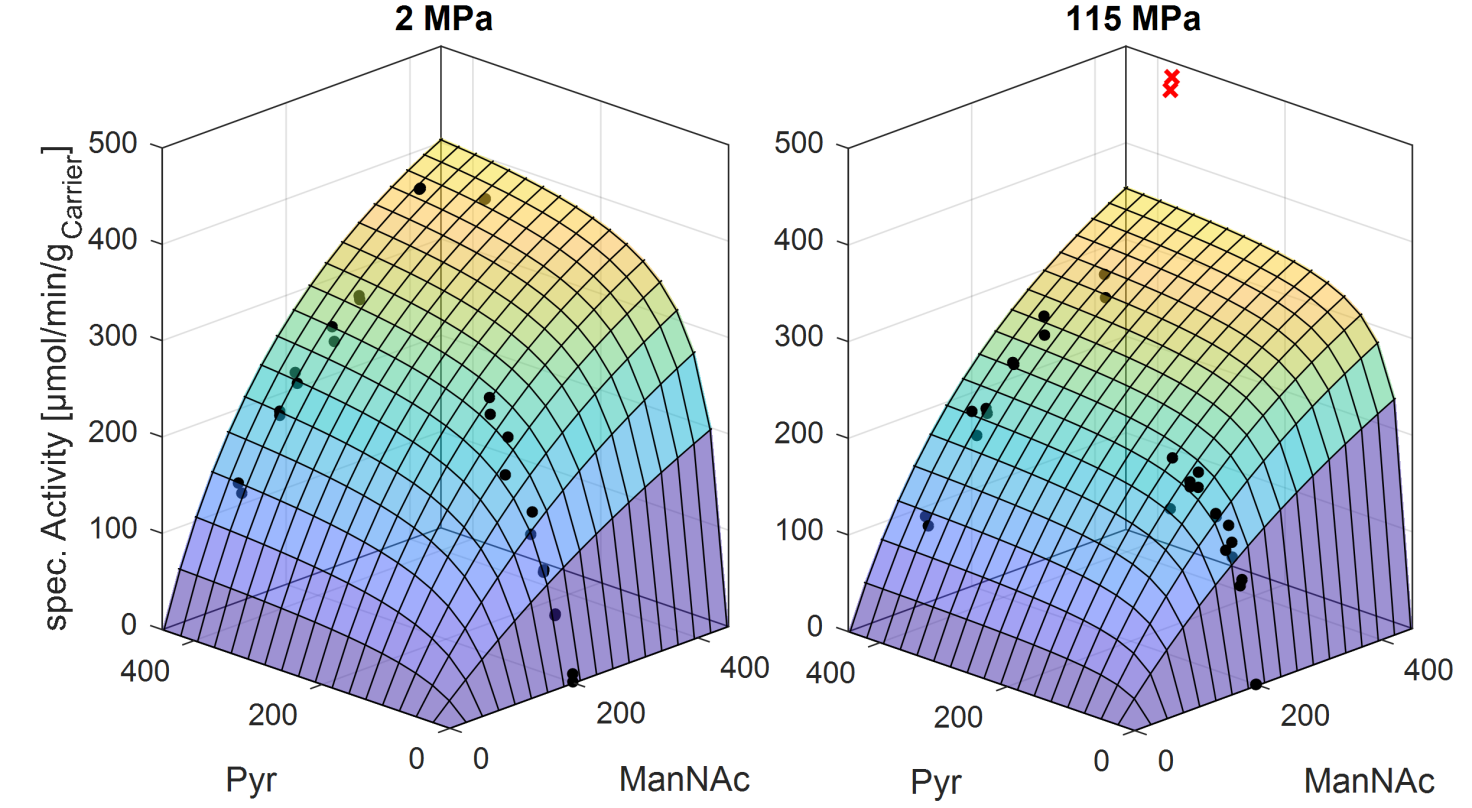
Measured kinetics of the aldolase when varying the pyruvate and ManNAc concentration (given in mM) at different pressures. Reaction conditions: 40 °C, flow rate: 1.5–2 mL/min, reactor volume: 0.70 mL, 100 mM potassium phosphate buffer (pH 7.50), 179.80 mg particles loaded with aldolase. Immobilisate was pestled.

Kinetic studies of the immobilized aldolase show an increase in affinity of pyruvate towards the enzyme (Figure 11). The calculated *K*_M_ values are 91 ± 45 mM and 37 ± 10 mM at 2 MPa and 115 MPa, respectively. A volume change for the binding of pyruvate to the enzyme of 20 mL/mol was calculated from the change in the Michaelis–Menten constant. The calculated kinetic parameters are listed in Table 5. Since Neu5Ac is acidic, 200 mM buffer solution with the addition of K_2_HPO_4_ was used to neutralize the solution to pH 7.2. Using the Haldane relation, the calculated equilibrium constants were 27 L/mol and 48 L/mol at 2 MPa and 115 MPa, respectively.

**Table 5:** Calculated kinetic parameters at ambient and high pressure. Rate expression adopted from Groher et al. [8].

Forward reactions	2 MPa	115 MPa

a_sp,max_ [U/g_carrier_]	650 ± 150	630 ± 130
*K*_M,ManNAc_ [mM]	230 ± 110	320 ± 120
*K*_M,Pyr_ [mM]	91 ± 45	37 ± 10

Backward reactions	2.5 MPa	93 MPa

*K*_M,Neu_ [mM]	650 ± 300	365 ± 260
a_sp,max_ [U/g_carrier_]	743 ± 230	403 ± 170

### Circulation reactor

**Figure 12:**
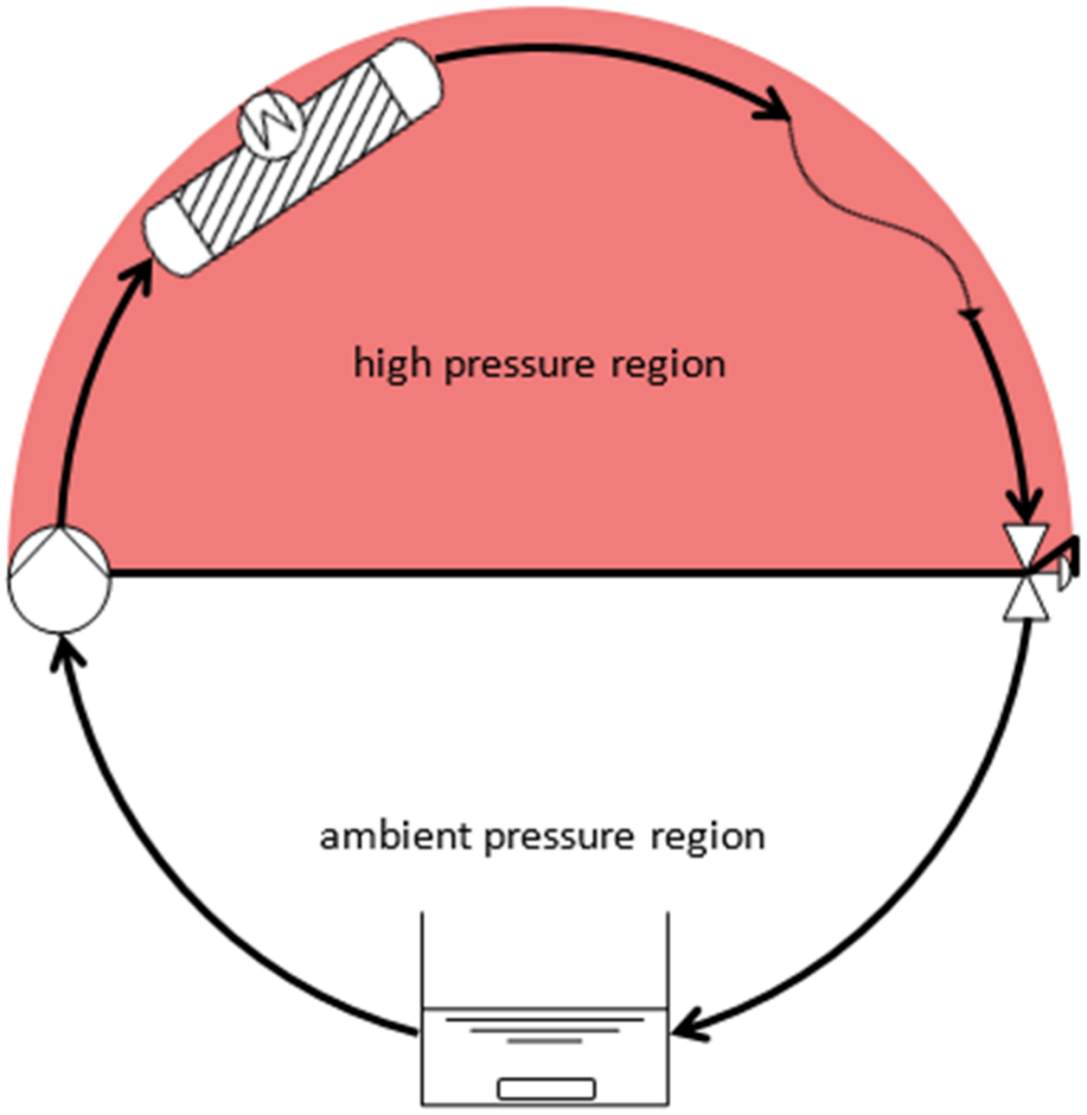
Circular reactor, vessel mixing was achieved with a magnetic stirrer and samples were taken directly from the vessel.

If the position of the equilibrium is to be investigated, high residence times and small flow rates are needed. Since the pressure drop across a capillary depends on the flow rate, it was not possible to build up sufficient pressure when investigating the equilibrium. For this reason, the fixed-bed reactor was changed into a circular reactor (Figure 12). In this set-up the flow rate can be set (almost) freely to achieve the desired pressure (mixing time is affected when the flow rate is low).

### Position of equilibrium

The position of the equilibrium was determined using the circular reactor. The ratio of product and substrates was calculated for each sample and converged to the equilibrium constant under the given conditions.

**Figure 13:**
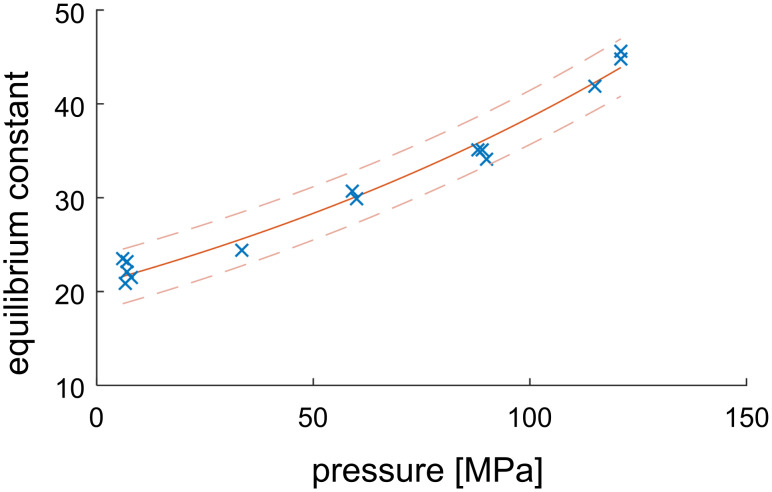
Aldolase: Change of the equilibrium constant at different pressures. Starting concentrations were varied: 100 mM with 250 mM and 100 mM with 100 mM of ManNAc and pyruvate, respectively. Conditions: 41 °C, reaction time at least 12 h, total working volume 4 mL, flow rate 1.5 mL/min, 272.3 mg particles loaded with aldolase. The used regression is an exponential fit according to Equation 1.

The equilibrium constant for the first reaction (one-to-one) was insensitive to pressure. For the second reaction (aldolase), the calculated equilibrium constant is shown in Figure 13. Since this reaction step is a two-to-one-reaction, a reduction in molar volume was expected, resulting in a positive influence of pressure (principle of Le Chatelier). The change in volume was calculated as −16.0 ± 1.2 mL/mol.

**Figure 14:**
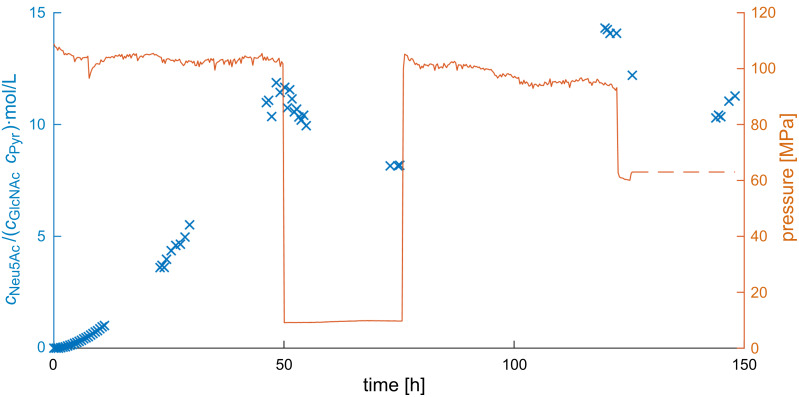
Progress curve of the circular reactor with both reactions at varying pressures. Starting conditions: 300 mM ManNAc and 300 mM Pyr, 100 mM potassium phosphate buffer at a pH of 7.50, 2 mL/min, total working volume: about 15 mL, 40 °C, 0.35 mL reactor volume, 1:9 (v/v) particles loaded with epimerase: particles loaded with aldolase, total mass: 85.4 mg particles.

Both immobilisates were added into one reactor and GlcNAc and Pyr were added as substrates to produce Neu5Ac. The resulting progress curve is shown in Figure 14. In order to measure changes in concentration with a high resolution, a high working volume was selected and the enzyme concentrations were reduced, leading to a slower conversion. Pressure was then varied, resulting in a change in substrate and product concentration, indicating that the system was sensitive to pressure.

Figure 14 shows the two advantages of the high-pressure circular reactor over a pressurized batch reactor: First, samples can be taken without the use of special pressure valves. Second, the pressure can be changed while the reaction is ongoing. If pressure is only changed up to 30 MPa, the operation can continue. If larger changes in pressure are required, the pump can be shut down, capillaries added or removed and the pump turned on again, resulting in a minimal downtime.

The combined reaction was, like the aldolase reaction, positively influenced by pressure. This was shown by the ratio of product to substrates (with the equilibrium constant *K* as the asymptote), as well as conversion, selectivity, and yield (Table 6). While the conversion, selectivity, and yield depend on the ratio of substrates, the equilibrium constant can also be used for other ratios.

**Table 6:** Changes in conversion, selectivity, and yield at different pressures.

Pressure [MPa]	Conversion [%]	Selectivity [%]	Yield [%]

9.6	57.9	81.9	47.4
60.8	60.4	82.7	50.0
95.0	63.9	84.7	54.1

^a^Same reaction conditions as in Figure 14.

## Conclusion

An epimerase and an aldolase were investigated to continuously produce *N*-acetylneuraminic acid under high pressure. Both enzymes were successfully immobilized with high stability and used to catalyze a reaction starting from *N*-acetyl-ᴅ-glucosamine and pyruvate. In addition, pressure up to 130 MPa was used to increase the conversion by 6.0%, the selectivity by 2.8%, and the yield by 6.7% (from 57.9% to 63.9%, 81.9% to 84.7%, and 47.4% to 54.1%, respectively). The increase in the value of the equilibrium constant with pressure suggests a reduction in molar volume. The circular reactor setup allowed for easy sampling and enabled the analysis of the resulting progress curve. The findings for the epimerase indicate that some inconspicuous reactions, such as the inhibition by pyruvate, can be influenced by pressure. In both reactions pyruvate showed increased affinity towards the investigated enzymes. Given the metabolic importance of pyruvate [33–35], it may be of interest to test different pyruvate converting enzymes if this trend is confirmed.

## Experimental

### Methodology

#### Genes and expression strains

The gene of the epimerase (*N*-acyl-ᴅ-glucosamine 2-epimerase, EC 5.1.3.8) from *Pedobacter heparinus* was ordered as codon optimized gBlocks gene fragment (Integrated DNA Technologies, Leuven, Belgium). The gene for the aldolase (*N*-acetylneuraminate lyase, EC 4.1.3.3) was amplified from the genomic DNA of *Escherichia coli* K12. The genes of the enzymes were cloned into the expression vector pETDuet-1™ (Novagen^®^, Merck KGaA, Darmstadt, Germany). For expression *E. coli* BL21 (DE3) strains were used.

#### Enzyme immobilization

For analysis of enzyme immobilization, six different carriers with different properties of a screening kit were used (ECRKIT1, Purolite Lifetech, Duisburg, Germany). Three carriers that bind the enzyme through absorption (ECR1030M, ECR8806F, ECR1090M), two carriers with epoxy functional groups for covalent immobilization (ECR8204F, ECR8806F), and one amino-functionalized carrier for covalent immobilization (ECR8309F). For all immobilizations, a 20 mM sodium phosphate buffer with a pH of 7.4 was used as immobilization buffer. All filtration steps were executed using a membrane pump (Membrane pump ME 2C NT, Vacuubrandt GMBH & Co. KG, Wertheim, Germany) with bottle topper filter (Nalgene™, Thermo Fisher Scientific GmbH, Schwerte, Germany), and membrane filters (3 µm) (Sartorius AG, Göttingen, Germany). The carriers were equilibrated with immobilization buffer at a carrier to buffer ratio of 1:1 (w/v). For the amino methacrylate carrier (ECR8309F), a further step of activation was performed with 2% glutaraldehyde solution 1:4 (w/v). After addition of the 2% glutaraldehyde solution, the mixture was incubated for 1 h at room temperature under slow rotation at 8 rpm using a sample mixer (MXIC1 sample mixer, Dynal Biotech Ltd., Bromborough, UK). Afterwards, the activated carrier was filtered and carefully washed with immobilization buffer. For immobilization, the buffer of purified enzymes (150 mM imidazol, 300 mM NaCl, 50 mM sodium phosphate buffer pH 7.4) was exchanged to the immobilization buffer using ultracentrifuge units with 10 kDa Cut-off (Sartorius Vivaspin™, Göttingen, Germany). The unbuffered enzymes were mixed with the different carriers (epimerase: 89.5 mg_enzyme_/g_carrier_, aldolase: 83.9 mg_enzyme_/g_carrier_) and incubated at 25 °C under slow rotation at 8 rpm using a sample mixer (MXIC1 sample mixer, Dynal Biotech Ltd., Bromborough, UK). After 18 h the rotation of the immobilization with the epoxide-functionalized carrier (ECR8204F, ECR8806F) was stopped and incubated for further 20 h at 25 °C. The immobilization process for the other carrier was stopped after 18 h (ECR8309F) and 24 h (ECR1030M, ECR8806F, ECR1090M). The carriers with immobilized enzymes were filtered and the filtrate was collected for protein quantification. Afterwards, the carriers were washed twice with immobilization buffer containing 0.5 M NaCl 1:1 (w/v) and three times with immobilization buffer 1:1 (w/v). They were stored afterwards refrigerated at 6 °C.

#### Activity assays

To compare the activities of both enzymes, a standard activity assay was used for the free and immobilized enzymes. For the epimerase, the reaction conditions were 100 mM Tris, pH 8, 40 °C, 100 mM *N*-acetyl-ᴅ-glucosamine, 1 mM adenosine triphosphate, 1 mM MgCl_2_, and 10 g/L immobilized enzyme or 2.5 mg/L free enzyme, respectively. For the aldolase, the reaction conditions were 100 mM Tris, pH 8, 40 °C, 100 mM *N*-acetyl-ᴅ-mannosamine, 250 mM pyruvate, and 50 g/L immobilized enzyme or 100 mg/L free enzyme, respectively.

#### Repetitive batch study

Reusability was analyzed in 2 mL micro reaction tubes, using 10 mg immobilized epimerase (in triplicate) or 70 mg immobilized aldolase (in duplicate), respectively. The reactions were started by adding 1 mL substrate solution, run for 30 min, and analyzed for product formation. Afterwards, the remaining substrate was removed, and the carriers were washed twice with 100 mM Tris pH 8, and used for the next cycle or stored at 6 °C for the next experiment. For each enzyme, 50 repetitive batches were analyzed.

#### Stability study

For stability investigations, samples (10 mg immobilized epimerase or 50 mg immobilized aldolase) in 2 mL micro reaction tubes were stored under four different conditions: Storage with residual moisture (immobilized enzyme after filtration under vacuum) at 6 °C. Storage in 20 mM sodium phosphate buffer, pH 7.5 at 6 °C and 40 °C, respectively and storage at 40 °C while shaking at 1000 rpm. Enzyme activity was measured at regular intervals over the storage period. For each measuring point, the initial activity was analyzed using a standard activity assay.

#### High-performance liquid chromatography (HPLC)

For quantification of the product *N*-acetylneuraminic acid, an Agilent HPLC system connected with a variable wavelength detector at 210 nm was used. Separation was realized with a Nucleogel Sugar 810H column (Macherey Nagel, Düren, Germany). The injection was set to 10 µL and compounds were eluted with an isocratic flow of 0.1% phosphoric acid at 30 °C. The retention order was *N*-acetylneuraminic acid (8.1 min), pyruvate (9.5 min), and *N*-acetylglucosamine (11.1 min).

#### Enzymatic quantification of *N*-acetyl-ᴅ-mannosamine

The quantification of ManNAc for the epimerase activity was realized with an enzymatic quantification assay as described in Klermund et al. using *N*-acylmannosamine 1-dehydrogenase (ManDH, EC. 1.1.1.233) [36]. The assay was performed in 100 mM Tris-HCl pH 8 containing up to 0.2 mM ManNAc, 2 mM NAD, and 0.05 mL ManDH solution with 3 kU/mL. After starting the reaction, the mixture was incubated at room temperature for 30 minutes. The resulting NADH concentration was measured with an Eppendorf spectrophotometer at 340 nm.

#### High-pressure set-up

An HPLC pump (Nexera X2 LC-30AD) by Shimadzu Deutschland (Duisburg, Germany) was used in order to generate a steady flow. All given pressures were measured by the pump. For the reactor, an emptied UHPLC column (length 50 mm, ID 3 mm) by ISERA GmbH (Düren, Germany) was filled with immobilized enzymes and pressure was built up via capillaries with a smaller inner diameter (50 µm).

If the position of the equilibrium was investigated, high residence times were required, resulting in low flow rates and pressure built-up. To circumvent this bottle neck, a circular reactor was designed. A flow rate can be chosen in order to achieve the desired pressure (usually 1.7–2 mL/min). Another advantage is the reduction of film diffusion on the carriers.

A key advantage of this setup is that sampling and reaction at high pressure can occur simultaneously and progress curves can be measured in one reactor. Prior publications investigating high-pressure batch reactors required to conduct several experiments and stop the reaction at different times [22,37]. Moreover, the circular reactor allows for a change in pressure via the back pressure regulator. A magnetic stirrer was used to mix the fluid in a vessel from which the pump draws its feed.

### Chemicals

All compounds were ordered from Biosynth Carbosynth (United Kingdom) and used without further purification. Buffer preparation: potassium phosphate buffer: 5.3 mL of 0.2 M potassium dihydrogenphosphate (KH_2_PO_4_) with 94.7 mL of 0.2 M potassium hydrogenphosphate (K_2_HPO_4_) in 100 mL water resulting in 200 mL of 100 mM solution. The pH was measured and afterwards adjusted to pH 7.50 or 8.00 by adding more potassium dihydrogen- or monohydrogenphophate solution. 1 M Tris buffer: 121.14 g tris(hydroxymethyl)aminomethane was dissolved in 800 mL H_2_O, the volume was filled up to 1 L with H_2_O, and the pH value adjusted with HCl.

### Analytics

For HPLC analysis the method according to Zimmermann [5] was used as a starting point, resulting in the use of a Eurocat H type (KNAUER Wissenschaftliche Geräte GmbH (Berlin, Germany)) in an HPLC system (0.8 mL/min, column temperature 65 °C, 55 °C for refraction index, 5 mM H_2_SO_4_ as eluent). The retention order was *N*-acetylneuraminic acid (14 min), pyruvate (15.5 min), *N*-acetyl-ᴅ-mannosamine (19 min), and *N*-acetyl-ᴅ-glucosamine (20 min).

### Enzymatic bed

The packing of the enzymatic bed was achieved via sedimentation of the particles. First, the reactor was filled with buffer solution. Then, a slurry of particles was prepared in buffer solution (10 or 100 mM KP_i_ buffer at pH 7.5) and taken up using a syringe. The syringe was then placed on top of the reactor forming a water bridge and allowing the particles to sediment. Once the reactor was filled, it was shaken to allow the bed to settle and refilled, if needed. Once the bed was packed, buffer solution was pumped through to compress the material. The reactor was then opened and new particles were added until the whole space was occupied.

### Residence time distribution

In order to verify that this method yields similar packed beds, the residence time distribution (RTD) was measured and compared. The pump, autosampler, and refraction index (RI) detector were used to measure the RDT (HPLC 1100er Series by Agilent). Five µL of a 10 mM buffer were used as a tracer with an injection rate of 1 mL/min, resulting in a rapid injection. The flow rate was set to 0.1, 0.35, and 0.5 mL/min. The RI was used to measure the refraction approximately twice a second.

The tracer was injected via an autosampler and measured using a RI detector (KNAUER Wissenschaftliche Geräte GmbH, Berlin, Germany). At a flow rate of 0.35 mL/min, a mean residence time of 1.5 ± 0.01 min was calculated. The mean residence time of the system itself needs to be considered and was determined to be 0.801 ± 0.003 min.

**Figure 15:**
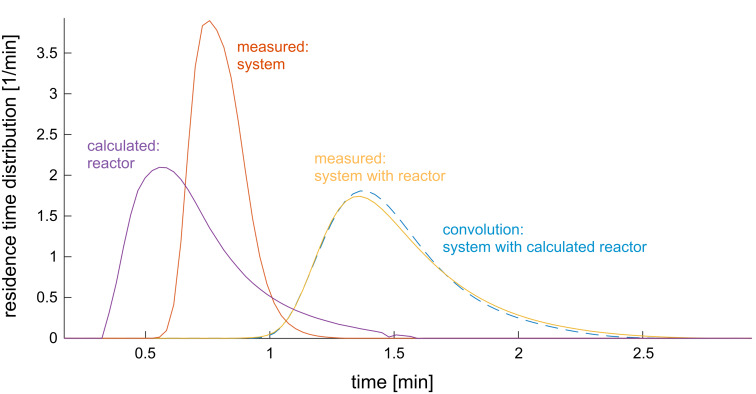
Residence time distributions of the stand-alone system and the reactor integrated into the system. Flow rate: 0.35 mL/min, reactor volume: 0.35 mL, filled with 74 to 80 mg of particles, 5 µL of 10 mM potassium phosphate buffer was used as tracer and injected with a speed of 1 mL/min.

The residence time distribution of the reactor was calculated assuming that the cumulative distributions are additive with respect to time. The obtained distribution of the reactor was convoluted with the distribution of the system and a result similar to the measured distribution of both was obtained (Figure 15).

The mean residence time was 0.7 min (as opposed to 1 min, obtained by dividing the whole reactor volume by the flow rate) when particles with the original size distribution were used. Since the diameter of the original particles is 0.5 mm and the inner diameter of the reactor is only 3 mm, wall effects occur [38–39].

When pestled particles were used, the mean residence time was 0.66 min by pumping 0.21 mL/min through a reactor volume of 0.21 mL. When using pestled particles, the residence time distribution is assumed to be narrow because the residence time distribution (RTD) of the system and of the whole setup are similar in shape and just shifted in time. Integration was conducted using Matlab 2017a and 2018a using the trapz function. The given values for τ do not account for the porosity of the packed bed. They are calculated via Equation 2


[2]

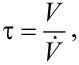



with *V* as the volume of the empty reactor and 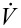 as the volumetric flow rate.

### Kinetics

**Figure 16:**
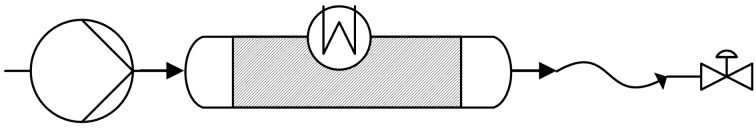
Reactor set-up (left to right): UHPLC pump, heated fixed-bed reactor, capillaries (ID: 25 µm or 50 µm, length: 30 cm), back pressure regulator (up to 30 MPa).

To investigate the influence of pressure on the selected reaction, a continuously operated fixed-bed reactor filled with immobilized enzyme was used (Figure 16). By setting a high flow rate, the determination of the reaction rate is possible via initial rate measurement. The pressure was built up using capillaries with small inner diameters (25 µm and 50 µm according to the law of Hagen–Poiseuille [40]).

The fixed bed reactor was utilized to investigate the reaction kinetics. The RDT of the fixed bed reactor was calculated following Equation 3 and used to calculate the mean residence time according to Equation 4.


[3] $$E\left(t\right)=\frac{S\left(t\right)}{\int S\left(t\right)dt},$$

[4] $$\bar{t}=\int t\cdot E\left(t\right)dt,$$

with *E* as the residence time distribution, *S* as the signal, and 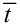 as the mean residence time.

The RDT was measured by placing the reactor into an HPLC, replacing the regular separation column. Hereby, the mean residence time of the reactor was calculated to be 66% of the quotient of reactor volume and flow rate.

When kinetic parameters were calculated by regression, the error given corresponds to the 95% confidence interval. All particles were pestled to remove potential diffusion limitations. All experiments were conducted at 40 °C and in potassium phosphate buffer. Since the pump is intended for UHPLC applications, a mixing chamber was installed for up to four eluents. In this study, the mixing chamber was used to change the concentration of the substrate to measure the reaction rate. A UV detector was used to ensure the homogeneity of the fluid generated by the mixing chamber. The order of concentrations was randomized to avoid systematic carry over to the next experiment.

The reaction rate (*v*) was calculated according to Equation 5, using the product concentration (*c*_ManAc_), the hydraulic residence time (τ), and the volume fraction (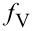).


[5] $$v=\frac{c_{\text{ManNAc}}}{f_{\text{V}}\cdot \tau }$$

The volume fraction was determined to be 0.66 for pestled particles (as shown in the section about residence time distribution).

### Equilibrium

Equation 1 describes the relationship between the equilibrium constant, change in molar volume, and pressure and was already applied in high-pressure investigations of enzymes [41]:


[1] $$K\left(p\right)=K\left(\text{0 MPa}\right)\cdot \exp\left\{-\frac{\Delta V}{RT}\cdot p\right\},$$

with *K* as the equilibrium constant, Δ*V* as the change in molar volume, *R* as the ideal gas constant, *T* as temperature, and *p* as pressure. Equation 1 was also used for the pressure dependency of the inhibition constant.

Conversion is calculated according to Equation 6 via a closed mass balance with *c*_GlcNAc_(0) = *c*_GlcNAc_(t) + *c*_ManNAc_(t),


[6] $$X\left(t\right)=\frac{c_{\text{ManNAc}}\left(t\right)}{c_{\text{GlcNAc}}\left(t\right)+c_{\text{ManNAc}}\left(t\right)}.$$

For a reaction with different products or a sequence of reactions, selectivity is calculated using the product concentration following Equation 7:


[7] $$S\left(t\right)=\frac{c_{\text{Neu5Ac}}\left(t\right)}{c_{\text{ManNAc}}\left(t\right)+c_{\text{Neu5Ac}}\left(t\right)}.$$
